# Phosphorescent Organic Light-Emitting Devices: Working Principle and Iridium Based Emitter Materials

**DOI:** 10.3390/ijms9081527

**Published:** 2008-08-26

**Authors:** Stefan Kappaun, Christian Slugovc, Emil J. W. List

**Affiliations:** 1 NanoTecCenter Weiz Forschungsgesellschaft mbH, Franz-Pichler-Straße 32, 8160 Weiz, Austria; 2 Institute for Chemistry and Technology of Materials, Graz University of Technology, Stremayrgasse 16, 8010 Graz, Austria; 3 Institute of Solid State Physics, Graz University of Technology, Petersgasse 16, 8010 Graz, Austria

**Keywords:** Organic light-emitting devices (OLEDs), electroluminescence, fluorescence, phosphorescence, phosphorescent polymers

## Abstract

Even though organic light-emitting device (OLED) technology has evolved to a point where it is now an important competitor to liquid crystal displays (LCDs), further scientific efforts devoted to the design, engineering and fabrication of OLEDs are required for complete commercialization of this technology. Along these lines, the present work reviews the essentials of OLED technology putting special focus on the general working principle of single and multilayer OLEDs, fluorescent and phosphorescent emitter materials as well as transfer processes in host materials doped with phosphorescent dyes. Moreover, as a prototypical example of phosphorescent emitter materials, a brief discussion of homo- and heteroleptic iridium(III) complexes is enclosed concentrating on their synthesis, photophysical properties and approaches for realizing iridium based phosphorescent polymers.

## 1. Introduction

Since the first observation of organic electroluminescence (i.e. the generation of light by electrical excitation of an organic material) in the 1960s by applying hundreds of volts to an anthracene single crystal [1], in the recent decades the field of organic electronics has progressed enormously [2]. Boosted by the pioneering work of Tang and VanSlyke and the resulting worldwide activity in numerous research groups [3], the advances in the fields of device science, device fabrication as well as chemistry, physics and materials science have evolved organic light-emitting device (OLED) technology to a point where it is now an important competitor to liquid crystals and liquid crystal displays (LCDs) [4–8]. Consequently, the interest in OLED technology has been impressive and first commercial products based on small molecules and conducting polymer films are already available. The OLED market is still growing and a large expansion in market penetration has been forecasted for the next years [2]. The tremendous interest in OLEDs and displays made from these devices is especially caused by technological aspects such as low costs, the ease of fabrication using standard techniques (e.g. vacuum deposition or solution processing) [9–13], the possibility of realizing flexible or large-area displays, their use in lighting applications and the variety of organic materials providing emission wavelengths that cover wavelengths from the ultraviolet to near infrared [14–16].

Although OLEDs already meet the requirements for some practical applications in, e.g., portable electronics like cellular phones or digital cameras [5] with operating lifetimes up to 100,000 hours [16], the intrinsic limits of organic light-emitting devices have not been reached yet [17]. Therefore, further scientific efforts devoted to the design, engineering and fabrication of OLEDs also considering charge carrier injection, spin effects, interfaces, quenching processes, morphology changes or light extraction have to be undertaken in order to provide a step forward to the complete commercialization of this technology [2]. Because fundamental understanding of the working principle of OLEDs is the crucial prerequisite for the further development of OLED technology, the basic processes occurring in electroluminescent devices are briefly reviewed in this work. Aside from device physics, also the design and synthesis of novel emitter materials plays an important role in commercialization of this technology. Therefore, the concepts of fluorescent and phosphorescent emitter materials are herein discussed putting special focus on phosphorescent iridium complexes (as prototypical example of dopants suitable for realizing high-performance electroluminescent devices).

## 2. The working principle of OLEDs

### 2.1. Small molecule OLEDs (smOLEDs) and polymeric light-emitting devices (PLEDs)

Conventional light-emitting devices commercially available today are mostly made from inorganic crystals such as GaAs or GaN. Although these devices work remarkably well for many applications, their use in large area displays or on flexible substrates is limited or even impossible because of the brittle crystals grown by expensive epitaxial methods [18]. As a consequence, organic materials have soon be considered to be promising alternatives to inorganic semiconductors, however, the practical application of OLEDs was unrealistic due to high operating voltages greater than 100 V until the fundamental breakthrough of Tang *et al.* in 1987 [3]. The landmark achievement of a double-layered architecture consisting of a hole transporting layer and an emissive layer of aluminum quinolinolate (Alq_3_) gave electroluminescence around 1,000 cd m^−2^ at driving voltages of 10 V and is still the archetypical setup for OLEDs [19].

Since that time tremendous scientific progress has been made resulting in numerous small molecules suitable for applications in OLEDs (frequently referred to as “small molecule OLEDs”, smOLEDs). Because thin films of these emitter materials are usually prepared by vacuum deposition, the interest in organic electroluminescence has further been heightened by the description of electroluminescence from conjugated polymers providing the possibility of solution processing (frequently referred to as “polymeric light-emitting devices”, PLEDs) [20]. As a result of this progress, organic semiconductors have developed rapidly from a topic of research interest into a wide range of applications. In this context, considerable advances concerning materials chemistry (e.g. the use of phosphorescent materials to increase device efficiencies) [9], device fabrication and device optimization [21–24] have been made giving OLEDs based on fluorescent small molecules, phosphorescent small molecules as well as polymeric materials (PLEDs) meeting industrial specifications. Although processing techniques are different for smOLEDs and PLEDs, their working principle is basically the same as it is reviewed on the subsequent pages.

### 2.2. Single and multilayer OLEDs

**Figure 1.**
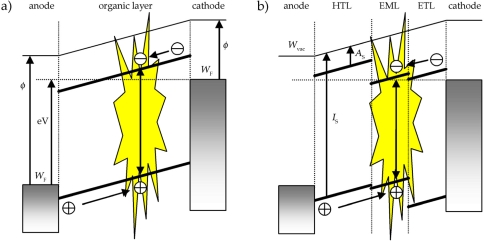
Energy level scheme of a a) single layer and b) double heterojunction OLED with an applied bias voltage *V* showing the vacuum energy level *W*_vac_, the Fermi levels *W*_F_ and work functions Φ of the metallic contacts (HTL = hole transport layer; EML = emissive layer; ETL = electron transport layer). The level offsets at the organic heterojunction are determined by the ionization energies *I*_S_ and electron affinities *A*_S_ [9].

Depending on device architecture, electroluminescent devices can be divided into single layer and multilayer OLEDs. Single layer OLEDs typically consist of a metal cathode with a low work function (e.g. Ca, Al, Ba), an organic emissive layer and a transparent anode [ e.g. indium tin oxide (ITO), which is a nonstoichiometric composite of SnO_2_ and In_2_O_3_] immobilized on a transparent carrier material such as glass or a flexible polymer [5, 25]. Under action of a driving voltage, electrons are injected from the cathode into the lowest unoccupied molecular orbital (LUMO) of the adjacent organic layer, while the anode injects holes into the highest occupied molecular orbital (HOMO) of the organic material. The electrons and holes move through the organic layer and recombine under the formation of an “exciton” capable of relaxing from its excited state to the ground state by emission of light (cf. Figure 1; here it should be noted that in organic materials mainly hopping transport in disordered materials occurs, whereas band transport mechanisms are typically discussed for inorganic materials) [9]. This process which is referred to as electroluminescence is the basic principle of OLEDs, nevertheless, some critical parameters have to be discussed.

**Figure 2.**
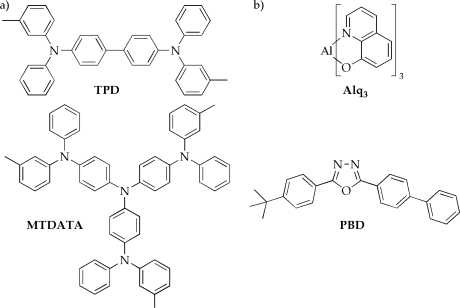
Commonly used a) hole transport (TPD = *N,N’*-diphenyl-*N,N’*-*bis*(*m*-tolyl)-1,1’-biphenyl-4,4’-diamine; MTDATA = *m*-methyl-*tris*(diphenylamine)triphenylamine) and b) electron transport materials (Alq_3_ = aluminum quinolinolate; PBD = (2-(4-biphenyl)-5-(4-*tert*-butylphenyl)-1,3,4-oxadiazol) used in OLEDs [5].

In electroluminescent devices the recombination zone should be in the middle of the emissive layer, thus, an equally efficient injection but also equal mobility of electrons and holes in the organic material are necessary. However, balanced charge injection or carrier mobility is usually not observed in the simple device architecture of single layer OLEDs resulting in decreased efficiencies because of exciton quenching processes close to the electrodes or non-radiative recombination of charges at the electrodes (dark current) [9]. These problems can be overcome by the incorporation of additional layers giving multilayer OLEDs. In these devices the emitter is sandwiched between hole and electron transport layers (cf. Figures 1 and 2) providing facilitated charge injection and enhanced recombination of electrons and holes in the emissive layer shifting the active zone roughly to the middle of the OLED structure. Along this line, state-of-the-art OLEDs have up to five layers, containing charge- and exciton blocking layers being essential for excellent device performance. Additionally, it should be mentioned that also doping of the transport levels can readily be used for increasing the efficiencies of the corresponding light-emitting devices [5–7, 9–13].

Multilayer devices can be built up either by vacuum sublimation or from solution. While vacuum deposition offers the clear advantage that multilayer structures can be easily realized also providing an additional purification step, sublimation processes suffer from a limited number of materials enduring thermal stress. Consequently, solution processing techniques have received considerable attention although serious problems such as orthogonal solvent systems have to be faced. The most convenient strategy to overcome this drawback has yet been proposed by Nuyken *et al.* applying precursor materials which are converted into insoluble networks by additional crosslinking steps [5,26].

### 2.3. Fluorescent and phosphorescent materials in OLEDs

For efficient OLEDs electrons and holes should form excited states capable of recombining radiatively and without energetic losses. Assuming that balanced charge injection is possible and that all injected charges recombine, considerations on quantum statistics are crucial for the realization of devices exhibiting high efficiencies [14].

Singlet excitons are states with an antisymmetric spin and a total spin quantum number *S* = 0. The quantum mechanically allowed transition to the ground state gives rise to fluorescence within nanoseconds. Conversely, triplet states have an even symmetry with *S* = 1 whose transition to the singlet ground state is quantum mechanically forbidden resulting in phosphorescence with lifetimes in the microsecond to second regime. As a consequence of the corresponding multiplicities of the angular momentum states (i.e. *m_S_* = 0 for *S* = 0 and *m_S_* = –1, 0, 1 for *S* = 1) and the random nature of spin production in electroluminescent devices, simple statistics predicts that only 25% of the injected charges result fluorescence (from singlet states) whereas 75% give phosphorescence (from triplet states) in suitable device architectures. Thus, uncorrelated electrons and holes form triplet states with a threefold higher probability than singlet states [9]. Here it should be noted, that recent studies on spin statistics suggest variations in the singlet-to-triplet-ratios. These findings have also been confirmed by quantum mechanical calculations [11].

The ground states of most luminescent materials are singlet states and the vast majority of luminescent compounds exhibits only weak spin-orbit couplings rendering these small molecules and polymers fluorescent with negligible radiative rates from triplet states. Competing nonradiative processes (e.g. triplet-triplet annihilation or vibronic relaxation) effectively quench the phosphorescence of the associated excited states clearly limiting the maximum quantum efficiency achievable with fluorescent small molecules and polymeric materials [14].

Since the pioneering work of Baldo *et al.* showing that phosphorescent dyes (transition metal compounds with organic ligands or organometallic compounds) doped into appropriate host materials give improved OLEDs [27], considerable scientific efforts have provided numerous new materials for electrophosphorescent devices reaching impressive external quantum efficiencies. Although these devices usually require elaborated multilayer structures, the fast and efficient intersystem crossing (ISC) from excited singlet to light-emitting triplet states caused by strong spin-orbit couplings as well as the ability of triplet harvesting allow theoretically quantum efficiencies of 100% [14, 25]. Today, a huge number of phosphorescent dyes are used in phosphorescent OLEDs utilizing different metal complexes containing transition metals such as iridium, platinum, osmium, ruthenium, etc. [28–30]. These transition metal complexes definitely exhibit a series of very desirable materials properties such as emission wavelengths covering the entire visible spectrum, high quantum yields and long lifetimes, however, severe concentration quenching is observed for most pure layers of phosphorescent dyes [31]. Consequently, phosphorescent dyes are usually blended into suitable host materials (small molecules or polymers) from which the excitation energy is transferred to the phosphorescent guest [14]. Thus, basic knowledge about the energy transfer processes is required for understanding the working principle of phosphorescent OLEDs.

### 2.4. Transfer processes in host materials doped with phosphorescent dyes

**Figure 3.**
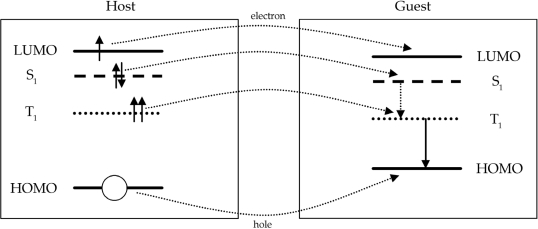
Simplified illustration of different transfer processes from the host to the guest. Direct electron/hole transfer from the host LUMO/HOMO to the guest LUMO/HOMO, Förster energy transfer between singlet states and Dexter energy transfer from the host to the guest triplet states are shown [14].

The transfer of excitation energy to the guest is promoted by three processes (cf. Figure 3), namely long-range Förster transfer of singlet excitons generated on the matrix to the guest, short-range Dexter transfer of singlet and triplet excitons generated on the host to the dopant and direct generation of singlet and triplet excitons on the guest (in this case the host solely acts as charge-transporting matrix). While for the Förster transfer a significant overlap of the emission spectrum of the matrix and the absorption spectrum of the guest is a crucial parameter, efficient Dexter transfer, on the other hand, requires that the energies of the singlet and triplet excitons on the host match the exciton energies on the guest. Also for direct carrier trapping on the phosphorescent dye a significant offset of the HOMO and LUMO energies of the host and guest material is necessary as schematically depicted in Figure 3 (here it should be noted that OLEDs are more efficient if the charges are trapped on the phosphorescent emitter. If Förster transfer is necessary, a loss in power efficiency is found because of host population processes) [14].

Irrespective which process results the excitation of the phosphorescent dye in an electrophosphorescent OLED, efficient triplet harvesting on the transition metal complex is only guaranteed if the T_1_–S_0_ energy of the guest is smaller than that of the host. Therefore, only a limited number of suitable host materials is known for most phosphorescent compounds also pointing out the need for new small molecules and polymeric materials capable of excitation of red, green and blue phosphors [14]. Although the processes giving electrophosphorescence are basically understood, large scientific efforts are ongoing studying the concomitant excitation routes in OLEDs. Excellent reviews on this topic have been published recently [14, 25].

### 2.5. Host materials for phosphorescent OLEDs

For the fabrication of efficient phosphorescent OLEDs, the transition metal complex is typically used as emitting guest in a host material to avoid triplet-triplet annihilation or quenching effects associated with the relatively long excited state lifetimes. Thus, the synthesis and characterization of adequate host materials are of significant importance and provide big challenges for chemists [32].

In general, a prerequisite for suitable host materials is that the triplet level of the host is larger than the triplet level of the phosphorescent guest for preventing undesired energy back transfer processes [32]. In addition to the match of the host and guest triplet states, also HOMO/LUMO levels permitting good charge injection as well as chemical and morphological stability of the host are critical parameters [33]. Furthermore, considerable overlaps between the absorption bands of the guest and the emission spectrum of the host are very desirable providing efficient Förster transfer [34]. Among the studied materials, only some classes fulfill the above mentioned boundary conditions. Especially carbazoles, polyphenylenes and fluorenes (small molecules as well as polymers; note that the T_1_-levels of, e.g., polyfluorenes are too low for being adequate host materials except for red triplet emitters) are of special interest because they are able to host red, green and even blue triplet emitters (cf. Figure 4) [35–49].

### 2.6. Advantages of OLEDs in display technology

The development of OLEDs in the last decades has shown that organic electroluminescence is a viable display principle for different fields of application [4]. Due to the very desirable materials properties of fluorescent and phosphorescent small molecules as well as semiconducting polymers (e.g. permitting vacuum deposition or solution processing) [18], the inexpensive manufacturing of pixilated displays has provided electroluminescent displays including the advantages of wide viewing angles, fast display response times in the microsecond regime, broad color ranges, high dark contrasts and thin display modules. Furthermore, OLEDs definitely provide numerous advantages such as high image quality or high power efficiency [4].

Even though it can be argued that liquid crystal or plasma displays exhibit similar or even superior properties especially concerning materials stability and longevity, the use of OLEDs offers some distinct new possibilities which are not accessible with other approaches. Among them, top and bottom emitting transparent OLEDs (TOLEDs) for, e.g., head-up displays, full color displays prepared from stacked red, green and blue subpixels, or OLED displays fabricated on flexible substrates are just few selected examples of promising fields of application of this technology [9].

In the context of further development of OLEDs for practical applications in display technology, particularly phosphorescent emitter materials have earned considerable attention because of the above discussed reasons (e.g. external quantum efficiencies, etc.). Not surprisingly, numerous reports can be found in literature focusing on the synthesis and characterization of novel iridium, platinum or osmium based complexes for realizing highly efficient phosphorescent OLEDs [28, 30, 50, 51]. Among these materials, phosphorescent iridium(III) complexes are anticipated to be the most promising candidates for practical applications. Therefore, we herein focus on this kind of phosphor briefly reviewing the different synthesis procedures and photophysical properties of homo- and heteroleptic iridium(III) complexes.

## 3. Phosphorescent iridium(III) complexes

### 3.1. Synthesis of iridium(III) complexes

The interest in iridium compounds (iridium(I) and iridium(III) species) has especially been boosted by the different fields of application ranging from very efficient catalysts to important emitter materials in OLEDs. Although the catalytic activity of iridium complexes has been well-known for a significant period of time [52], a new era of utilization has commenced its rapid development with the first reports on electrophosphorescent devices [27]. In particular, the very desirable materials properties such as reversible electrochemistry, adequate triplet lifetimes, synthetic versatility, color-tuning of the emission wavelengths by ligand modifications or the robust nature of many iridium(III) complexes render these compounds ideal candidates for the fabrication of highly efficient phosphorescent OLEDs [53–55]. Neutral electrophosphorescent iridium(III) compounds are definitely the most common phosphorescent dopants in OLEDs distinguishing two classes, namely homoleptic compounds containing three cyclometalating ligands and heteroleptic iridium complexes with two cyclometalating ligands and an ancillary ligand. Nevertheless intense studies on neutral iridium complexes are topic of worldwide scientific efforts, also charged derivatives receive more and more attention due to possible applications in light-emitting electrochemical cells (LECs) [56].

**Scheme 1.**
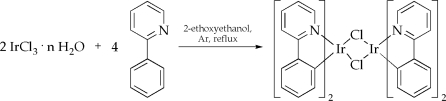
Preparation of the μ-chloro bridged precursor material di-μ-chloro-*tetrakis-*(κ^2^(*C^2^*,*N*)-2-phenylpyridine)diiridium(III) as reported by Nonoyama [57].

For the preparation of these compounds usually the “bridge-splitting” approach is used. In this synthesis strategy the cyclometalating ligand (e.g. phenylpyridine, benzoquinoline, 2-phenylbenzothiazole, etc.) is reacted with IrCl_37_·nH_2_O in 2-ethoxyethanol under inert atmosphere giving the corresponding air-stable μ-chloro bridged precursor material as reported by Nonoyama (cf. Scheme 1) [57]. Although this procedure is widely used, alternative protocols performing the cyclometalation reaction in trimethyl phosphate at significantly lower temperatures have been reported [58].

**Scheme 2.**
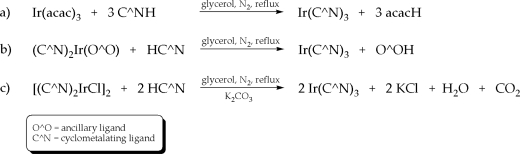
Different synthetic routes for the preparation of *tris*-cyclometalated iridium(III) complexes [55].

The first report on homoleptic *tris*-cyclometalated iridium(III) compounds was published in the early 1980s being observed as a byproduct of the synthesis of di-μ-chloro*tetrakis*(κ^2^(*C^2^*,*N*)-2-phenyl-pyridine)diiridium(III) [59]. Since that time and enforced by the excellent electroluminescent properties of e.g. Ir(ppy)_3_ (ppy = phenylpyridine) as well as the possibility of efficient color-tuning by simple changes or modifications of the cyclometalating ligand, numerous optimized protocols have been presented giving *tris*-cyclometalated complexes in acceptable yields. Among them, ligand-exchange reactions starting from Ir(acac)_3_, solvent-free procedures or approaches conducted at high temperatures of 170 – 195°C in the presence of silver triflates have been proposed (cf. Scheme 2) [55]. However, the harsh reaction conditions, comparable low yields and the number of byproducts [60] are considered to be significant drawbacks of homoleptic iridium complexes. Additionally, the rate of formation of the *facial* and *meridional* isomer was found to be influenced by the reaction conditions. It is worth noting that these coordination isomers exhibit different photophysical properties [61–63] and undergo isomerization processes in, e.g., working electroluminescent devices [55]. From aluminum quinolinolates it is well known that such effects give undesired changes of the device characteristics [64], therefore, this is considered to be another shortcoming of homoleptic iridium compounds.

In contrast to the difficulties with the preparation of *tris*-cyclometalated iridium complexes, an alternative synthesis approach is based on incorporating ancillary ligands such as acetylacetonates (cf. Figure 4), picolinates, triazolates, tetrazolates or quinolinolates [65–67]. Although the preparation of heteroleptic iridium(III) compounds starts, again, from the corresponding μ-chloro bridged precursor materials, the subsequent bridge-splitting step is clearly facilitated giving the products in better or even quantitative yields. However this is a distinct advantage, only few successful examples of heteroleptic iridium compounds with emission properties dominated by these ligands have been reported [65]. Thus, color-tuning is usually achieved by changing the nature of the cyclometalating ligand but, on the other hand, requires the synthesis of different precursors [55, 68]. Nevertheless this is considered to be a significant drawback of this approach, many heteroleptic iridium complexes bearing ancillary ligands exhibit very desirable materials properties such as high quantum yields even at room temperature or good thermal stabilities providing the possibility of vacuum deposition processes. As a consequence, they are frequently used as phosphorescent dopants in OLEDs [68–70].

### 3.2. Photophysical properties of iridium(III) complexes

Tuning of the photophysical properties of iridium(III) complexes has received considerable attention because of a series of practical applications such as flat-panel displays. Even though numerous organoiridium compounds have been reported giving efficient electroluminescence in the red [68], green [55] and blue spectral region [71–77], further scientific efforts are necessary to provide a general toolbox for realizing iridium compounds with absorption and emission characteristics tailored towards particular needs [53, 78]. In this context, numerous reports on the synthesis and photophysical characterization of novel iridium(III) complexes have been published [53, 55, 78], but also quantum mechanical calculations are becoming more and more important in the design of phosphorescent emitter materials [79–83].

The absorption and emission spectra of phosphorescent metal complexes are in general influenced by various parameters such as the valence electron configuration at the metal, the type of the electronic transitions or the correlation among lower lying electronic excited states [78]. Similar to fluorescent small molecules also the photophysical behavior of transition metal complexes can be properly described with molecular orbitals, however, the performance of accurate quantum mechanical calculations is far more complicated [79]. Nevertheless, it has been shown that frontier molecular orbitals are not equally delocalized in heavy metal complexes resulting in different electronically excited states [78].

Among them, metal-centered (MC) excited states are typically present in metal complexes with partially filled d shells at the metal center. The corresponding d-d transitions are Laporte-forbidden and, consequently, exhibit very low transition probabilities. Metal-to-ligand charge-transfer (MLCT) states involve electronic transitions from metal based d orbitals to a ligand centered π* antibonding orbital. Emissive MLCT states are particularly observed in d^6^ and d^8^ transition metal complexes and play, therefore, a major role in the photophysics of iridium(III) compounds. Intraligand (IL) π–π* excited states originate from electronic transitions of the ligand. If the metal perturbation upon coordination is minimized, their spectral properties often closely resemble the free ligand states. Finally, ligand-to-metal charge transfer (LMCT) excited states are occasionally observed in complexes with metal atoms in high oxidation states or in d^10^ complexes [28, 78, 79].

All these transitions determine the photophysical properties of transition metal complexes and can be used for the interpretation of experimentally observed spectra or prediction of absorption and emission characteristics of novel compounds [28, 78, 79]. Hence, this elementary knowledge of the described processes is necessary to rationalize the impact of ligand modifications on the photophysical properties of phosphorescent dyes or to design highly efficient materials for OLED applications.

**Figure 5.**
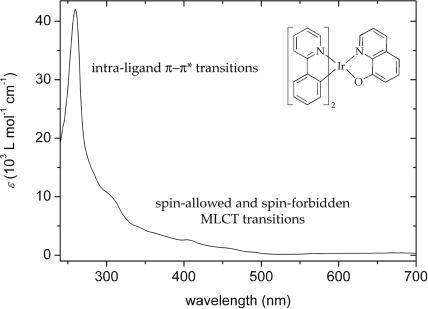
The absorption spectra of iridium(III) compounds typically show MLCT and π–π* transitions. Here the spectrum of *bis*(κ^2^(*C^2^,N*)-2-phenylpyridine)(κ^2^(*N,O*)-8-quinolinolate)iridium(III) is shown [67].

Along these lines, the absorption spectra of classical phosphorescent iridium(III) complexes typically display absorption bands with extinction coefficients between approximately 50000 and 6000 L mol^−1^ cm^−1^ (cf. Figure 5 as a typical example). The weak absorption features between 500 and 350 nm can be attributed to spin-allowed and spin-forbidden metal-to-ligand charge-transfer (MLCT) transitions, while the strong absorption bands peaking in the UV-region usually originate from intra-ligand (IL) π–π* transitions [14, 73].

**Figure 6.**
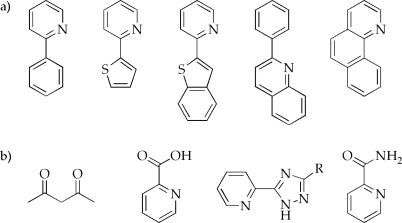
Commonly used a) cyclometalating and b) ancillary ligands for homo- and heteroleptic iridium(III) complexes [28, 53, 55, 78, 79].

After excitation of the iridium complex, the strong spin-orbit coupling induced by the metal center gives the formally forbidden triplet to singlet ground state transition a significant allowedness [14]. In this context, the energies of the lowest excited states play a major role as they can be tuned by adjusting the metal and ligand orbitals through substituent effects or *via* changing the ligand structures (some frequently used cyclometalating and ancillary ligands are shown in Figure 6). In other words, chemical modifications and/or complete alterations of the cyclometalating and/or ancillary ligands pave the way to very efficient emission color tuning but also provide the possibility of tuning the corresponding absorption characteristics towards particular needs [66, 67].

**Figure 7.**
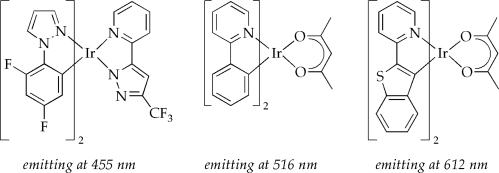
Selected examples of phosphorescent iridium complexes with emission maxima in the blue, green and red spectral region [68, 71].

Tremendous emission color-versatility has been achieved with iridium(III) luminophores applying the above described tuning procedures giving materials with a broad range of excited state lifetimes (from nanoseconds to microseconds; here it should be noted that a long lifetime will increase the probability of excited state quenching processes and increases diffusion of the excited state, which will in turn call for efficient concepts to confine the excitation in the emissive layer) and quantum yields approaching 100% [53]. As a consequence, phosphorescent iridium complexes have emerged as the most promising class for practical OLED applications and the number of new phosphorescent dyes with emission wavelengths covering the entire visible spectrum is still growing. Some selected examples of organoiridium complexes emitting in the blue, green and red spectral region are depicted in Figure 7. A more detailed summary of various iridium complexes and their photophysical characteristics can be found elsewhere [28, 53, 55, 68–79].

### 3.3. Polymeric phosphorescent materials

Even though many phosphorescent dyes are very promising candidates for realizing highly efficient OLEDs, for numerous phosphorescent small molecules serious problems such as the complexity of device fabrication or insufficient fine-dispersion of the dopant in the host material have been encountered. Especially phase separation is concerned as critical drawback, thus, covalent incorporation of the phosphorescent chromophores into polymers has received considerable attention.

**Figure 8.**
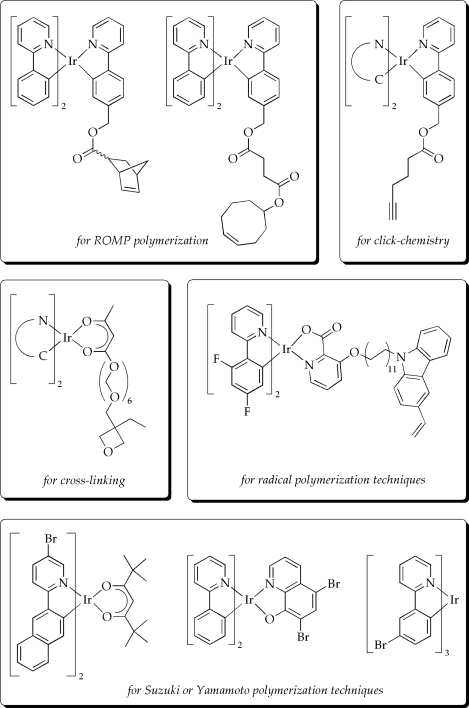
Selected examples of precursor materials for different polymerization techniques [84–102].

As a result, numerous concepts for the preparation of phosphorescent polymers have been proposed, among them utilization of ring-opening metathesis polymerization (ROMP), click-chemistry, the use of cross-linkable iridium complexes, grafting approaches or radical polymerization techniques [84–96]. In addition to these methods giving side-chain polymers, the incorporation of the phosphorescent material in the main-chain using various cross-coupling reactions is of particular interest for realizing solution processable emitter materials (some selected examples of precursor materials for the preparation of phosphorescent polymers are shown in Figure 8) [97–102]. Indeed, all these approaches have been demonstrated to overcome the shortcomings of phosphorescent small molecules and are, therefore, believed to contribute significantly to the further development of OLED technology.

## 3. Conclusions and Outlook

Herein the essentials of OLED technology have been reviewed putting special focus on the working principle of single and multilayer OLEDs as well as the use of fluorescent and phosphorescent emitter materials. After discussion of the advantages of phosphorescent dyes doped into suitable host materials and the corresponding energy transfer processes, the synthesis and photophysical properties of phosphorescent iridium(III) complexes have been enclosed. It has been shown that this class of materials exhibits photophysical characteristics readily tuned by changes of the cyclometalating and/or ancillary ligands, thus being ideal candidates for applications in, e.g., full color displays. Furthermore, different strategies for incorporating iridium complexes into polymers have been presented giving phosphorescent polymers with reduced phase separation in combination with facilitated device fabrication (e.g. solution processing).

The development of OLEDs has undoubtedly been empowered by different applications in display technology. As a consequence, tremendous scientific efforts have been devoted to this topic concentrating on the design, engineering and fabrication of OLEDs. Nevertheless, the intrinsic limits of OLED technology have not been reached yet. Hence, further progress in device physics and materials science is necessary to provide a step forward to the complete commercialization of this highly promising technology.

## Figures and Tables

**Figure 4.**
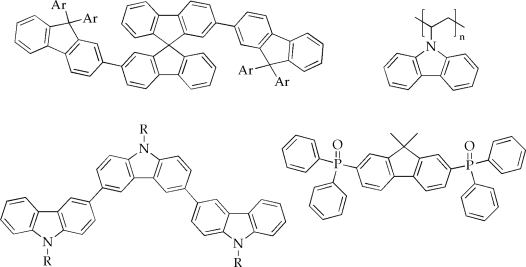
Selected examples of host materials for phosphorescent iridium(III) complexes [32–48].

**Figure 4.**
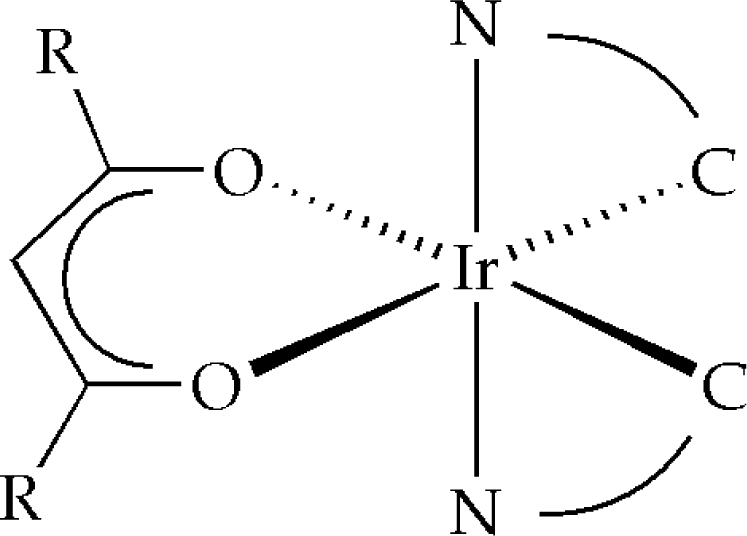
Schematic representation of a heteroleptic iridium(III) complex bearing an ancillary acac ligand [55].
